# Accelerating electron tomography reconstruction algorithm ICON with GPU

**DOI:** 10.1007/s41048-017-0041-z

**Published:** 2017-07-04

**Authors:** Yu Chen, Zihao Wang, Jingrong Zhang, Lun Li, Xiaohua Wan, Fei Sun, Fa Zhang

**Affiliations:** 1 Key Laboratory of Intelligent Information Processing, Institute of Computing Technology, Chinese Academy of Sciences, Beijing, 100190 China; 2 University of Chinese Academy of Sciences, Beijing, 100049 China; 3 School of Mathematical Sciences, University of Chinese Academy of Sciences, Beijing, 100049 China; 4 National Key Laboratory of Biomacromolecules, CAS Center for Excellence in Biomacromolecules, Institute of Biophysics, Chinese Academy of Sciences, Beijing, 100101 China; 5 Center for Biological Imaging, Institute of Biophysics, Chinese Academy of Sciences, Beijing, 100101 China

**Keywords:** Acceleration, Electron tomography, GPU, ICON, Missing wedge restoration

## Abstract

Electron tomography (ET) plays an important role in studying *in situ* cell ultrastructure in three-dimensional space. Due to limited tilt angles, ET reconstruction always suffers from the “missing wedge” problem. With a validation procedure, iterative compressed-sensing optimized NUFFT reconstruction (ICON) demonstrates its power in the restoration of validated missing information for low SNR biological ET dataset. However, the huge computational demand has become a major problem for the application of ICON. In this work, we analyzed the framework of ICON and classified the operations of major steps of ICON reconstruction into three types. Accordingly, we designed parallel strategies and implemented them on graphics processing units (GPU) to generate a parallel program ICON-GPU. With high accuracy, ICON-GPU has a great acceleration compared to its CPU version, up to 83.7×, greatly relieving ICON’s dependence on computing resource.

## Introduction

Electron tomography (ET) plays an important role in studying *in situ* cell ultrastructure in three-dimensional space (Yahav* et al*. 2011; Fridman* et al*. 2012; Rigort* et al*. 2012; Lučić* et al*. 2013). Combining with a sub-volume averaging approach (Castaño-Díez* et al*. 2012), ET demonstrates its power in investigating high-resolution *in situ* conformational dynamics of macromolecular complexes. Due to limited tilt angles, traditional ET reconstruction algorithms including weighted back projection (WBP) (Radermacher 1992), simultaneous iterative reconstruction technique (SIRT) (Gilbert 1972), direct Fourier reconstruction (DFR) (Mersereau 1976), iterative non-uniform fast Fourier transform (NUFFT) reconstruction (INFR) (Chen and Förster 2014), *etc*., always suffer from the “missing wedge” problem, which causes density elongation and ray artifacts in the reconstructed structure. Such ray artifacts will blur the structural details of the reconstruction and weaken the further biological interpretation (Lučić* et al*. 2005).

In recent years, many algorithms have been proposed to deal with the “missing wedge” problem. Some of them apply prior constrains to the reconstructed tomogram to compensate the missing wedge, such as filtered iterative reconstruction technique (FIRT) (Chen* et al*. 2016), discrete algebraic reconstruction technique (DART) (Batenburg and Sijbers 2011), and projection onto convex sets (POCS) (Sezan and Stark 1983; Carazo and Carrascosa 1987). These constraints include density smoothness, density non-negativity, density localness, *etc*. Others try to solve the reconstruction problem as an underdetermined problem based on a theoretical framework called “compressed sensing” (CS) (Donoho 2006). Compressed sensing electron tomography (Saghi* et al*. 2011, 2015; Goris* et al*. 2012; Leary* et al*. 2013) demonstrated certain success for the data with a high signal to noise ratio (SNR) (*e.g*., material science data or resin-embedded section data). To cope with the low SNR case (*e.g*., biological cryo-ET data, in which a low total dose of electron is used to avoid significant radiation damage), Deng* et al*. proposed iterative compressed-sensing optimized NUFFT reconstruction (ICON) by combining CS and NUFFT together (Deng* et al*. 2016). With a validation procedure, ICON not only restores the missing information but also measures the fidelity of the information restoration. ICON demonstrated its power in the restoration of validated missing information for low SNR biological ET dataset.

However, the convergence process of ICON is time-consuming. The huge computational demand has become a major problem for the application of ICON. The traditional solution to cope with the high computational cost has been the use of supercomputers and large computer clusters (Fernández* et al*. 2004; Fernández 2008), but such hardware is expensive and can also be difficult to use. Graphics processing units (GPU) (Lindholm* et al*. 2008) can be the attractive alternative solution in terms of price and performance. In this work, we developed the parallel strategies of ICON and implemented a GPU version of ICON, named ICON-GPU. Experimental results based on a Tesla K20c GPU card showed that ICON-GPU exhibits the same accuracy and a significant acceleration in comparison with the CPU version of ICON (ICON-CPU).

## Results and discussion

### Reconstruction precision

First, we evaluated the numerical accuracy of ICON-GPU using the root-mean-square relative error (RMSRE) *ɛ* as Eq. 1. To avoid dividing 0 when calculating the RMSRE, we first normalized the reconstructed slices into (0,1] using Eq. 2.

1 $$\varepsilon = \sqrt {\frac{{\mathop \sum \nolimits_{i = 1}^{N} \left( {\frac{{P{\text{norm}}_{i} - C{\text{norm}}_{i} }}{{C{\text{norm}}_{i} }}} \right)^{2} }}{N}} ,$$

where *N* is the size of one slice; $C{\text{norm}}$ is the normalized slice reconstructed by ICON-CPU; *C*norm_*i*_ is the value of the *i*th pixel in *C*norm; *P*norm is the normalized slice reconstructed by ICON-GPU; *P*norm_*i*_ is the value of the *i*th pixel in *P*norm.

2 $$P{\text{norm}} = \frac{{ P - { \hbox{min} }P}}{{{ \hbox{max} }P - { \hbox{min} }P}} + c,$$

where *P*norm is the normalized slice; $P$ is the originally reconstructed slice; min*P* is the minimum value of *P*; max*P* is the maximum value of *P*; *c* is a small constant to avoid 0 in *P*norm, in this work, *c* = 10^−7^.

**Fig. 1:**
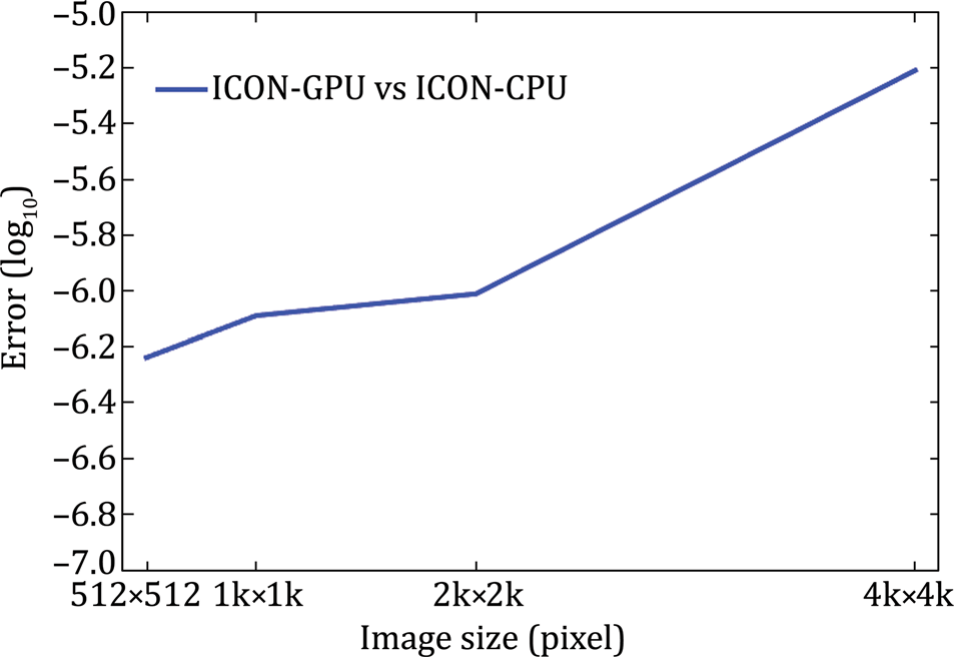
The RMSREs of ICON-GPU

The RMSRE of ICON-GPU increases slowly with the image size; they are in the range of $(6 \times 10^{ - 7} , \;4 \times 10^{ - 6} )$ yielding a reasonable numerical accuracy for the float format data (Fig. 1).

**Fig. 2:**
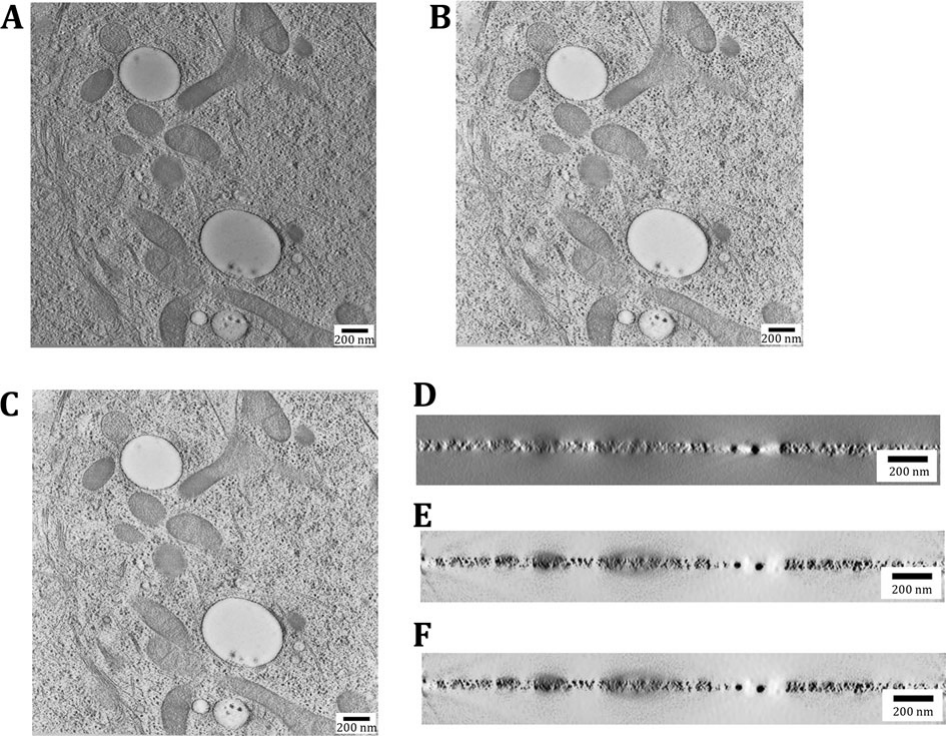
Evaluate ICON-GPU by investigating the reconstructed tomograms. **A**–**C** The *XY*-slices of the tomograms reconstructed by WBP, ICON-CPU, and ICON-GPU, respectively; **D**–**F** The *XZ*-slices of the tomograms reconstructed by WBP, ICON-CPU, and ICON-GPU, respectively

Then, we evaluated the reconstruction accuracy by investigating the reconstructed tomograms. The *XY*-slices reconstructed by ICONs (Fig. 2B, C) show better SNR than that by WBP (Fig. 2A), yielding a better contrast to discriminate the cellular ultrastructures. Besides, ICON-CPU and ICON-GPU are identical with each other and the normalized cross-correlation (NCC) between them is 1. The *XZ*-slices reconstructed by ICONs (Fig. 2E, F) are also identical with each other and the ray artifacts in ICONs are significantly reduced in comparison with WBP (Fig. 2D). To be noted that, to eliminate any suspicion on the gray-scale manipulation (which could enhance the visual advantage), all images were normalized and displayed based on their minimum and maximum value.

**Fig. 3:**
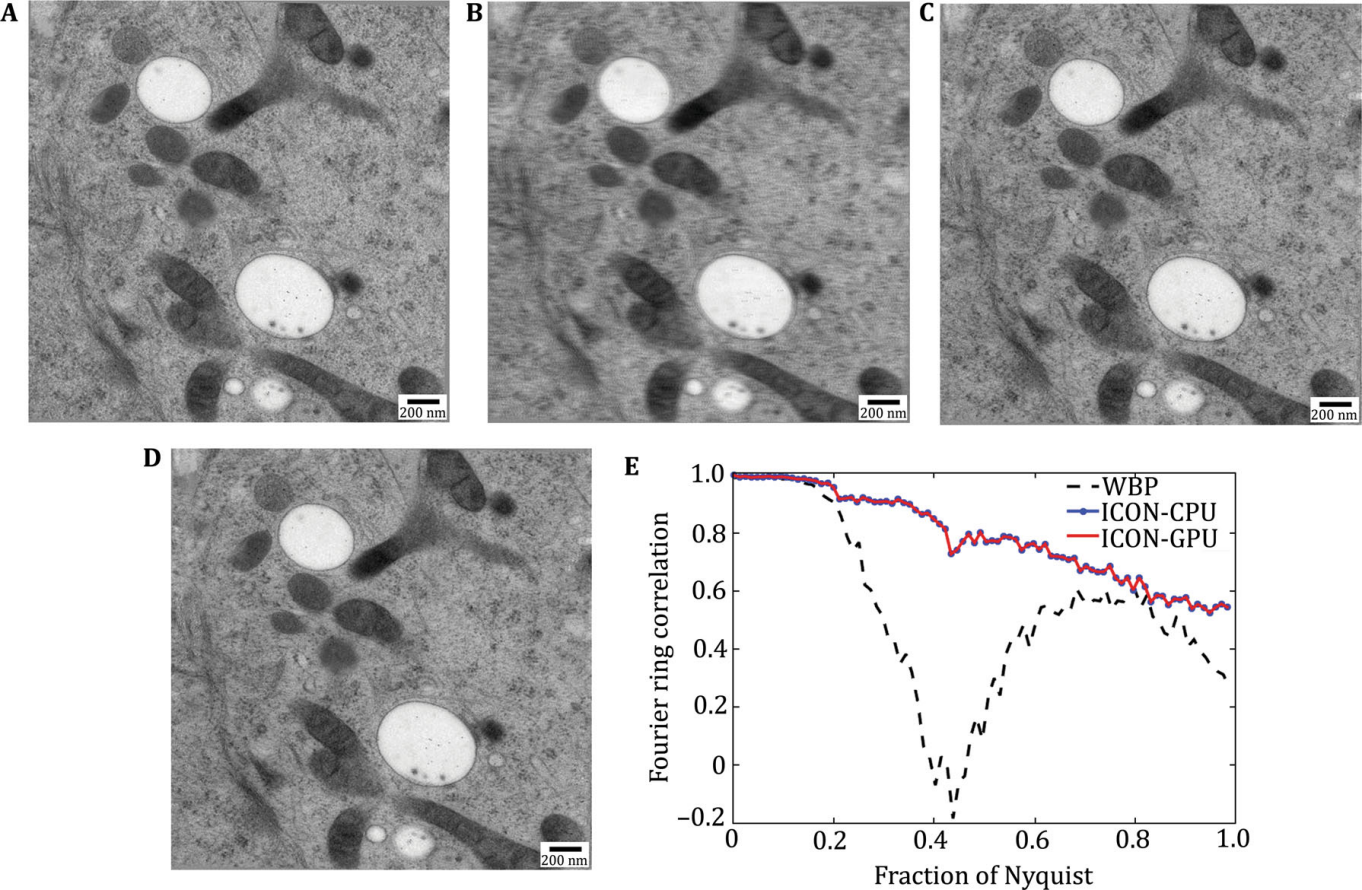
Evaluate ICON-GPU by the pseudo-missing-validation procedure. **A** The omit-projection (“Ground truth”); **B**–**D** The re-projections of the omit-tomograms reconstructed by WBP, ICON-CPU, and ICON-GPU, respectively; **E** The pseudo-missing-validation FRCs of WBP, ICON-CPU, and ICON-GPU

We further investigated the reconstruction accuracy by the pseudo-missing-validation procedure (Deng* et al*. 2016). Here, the −0.29° tilt (the minimum tilt) projection was excluded as the omit-projection (“ground truth”) (Fig. 3A). We re-projected the reconstructed omit-tomograms at −0.29°. The re-projections of ICONs (Fig. 3C, D) are identical with each other and the NCC between them is 1. The re-projections of ICONs are clearer in detailed structures and close to the “ground truth”, compared to that of WBP (Fig. 3B). Such visual assessments were further verified quantitatively by comparing the Fourier ring correlation (FRC) curves between the re-projections and the “ground truth”. The FRCs of ICONs coincide with each other, and they are better than that of WBP (Fig. 3E). The coincident FRCs of ICONs further demonstrate the accuracy of ICON-GPU from the perspective of restoring missing information.

### Speed up

**Fig. 4:**
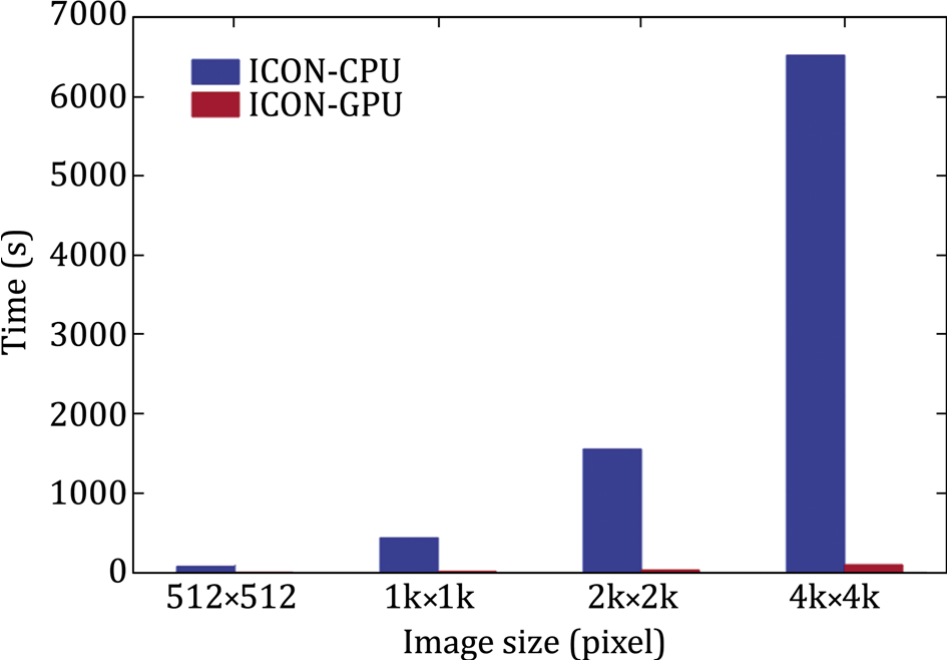
The comparison of time-consuming of ICON-CPU and ICON-GPU

We evaluated the acceleration of ICON-GPU by comparing the running time of reconstructing one slice under 200 iterations. We reconstructed the datasets with sizes of 512 × 512, 1 k × 1 k, 2 k × 2 k, 4 k × 4 k, respectively. The acceleration of ICON-GPU improves when the slice size increases (Fig. 4; Table 1). The maximum speedup is 83.7× in the reconstruction of a 4 k × 4 k slice. With the efficient acceleration, the reconstruction time of one 4 k × 4 k slice is reduced from hours to minutes, which greatly relieves ICON’s dependence on computing resource.

**Table 1:** The speedups of ICON-GPU compared to ICON-CPU

Image size	Speedup
512^2^	8.7×
1024^2^	41.5×
2048^2^	61.2×
4096^2^	83.7×

## Conclusions

In the present work, we analyzed the iterative framework of ICON and classified the operations of ICON reconstruction into three types. Accordingly, we designed parallel strategies and implemented them on GPU to generate a parallel program ICON-GPU. We tested ICON-GPU on a resin-embedded ET dataset of MDCK cell section. The RMSRE between ICON-GPU and ICON-CPU is about 10e^−6^, yielding a reasonable numerical accuracy of ICON-GPU compared to ICON-CPU. In addition, ICON-GPU has the same ability of restoring missing information with ICON-CPU. In addition, ICON-GPU has a great acceleration, up to 83.7× in the reconstruction of one 4 k × 4 k slice in comparison with ICON-CPU.

To be noted that, ICON-GPU can also run on multiple GPU system such as TIANHE-2, a supercomputer developed by China’s National University of Defense Technology, which is based on multi-core and many-core architectures (Liao* et al*. 2014).

The software package of ICON-GPU can be obtained from our website (http://feilab.ibp.ac.cn/LBEMSB/ICON.html or http://ear.ict.ac.cn).

## Materials and methods

### Iterative compressed-sensing optimized NUFFT reconstruction (ICON)

ICON is an iterative reconstruction algorithm based on the theoretical framework of “compressed sensing” and is designed to restore missing information caused by limited angular sampling (Deng* et al*. 2016). ICON is formulated as Eq. 3.

3 $$argmin \, \| \, Px \, \| _{{L_{0} }} , \, subject \, to: \| \, A^{h} WAx - A^{h} Wf \, \| _{{L_{2} }} < \varepsilon , $$

where *x* is the two-dimensional (2D) reconstructed slice; *W* follows INFR’s description (Chen and Förster 2014) and contains the weights that account for the non-uniform sampling in the Fourier space (similar to the ramp filtering in WBP); *A* is the projection operation, defined as a non-uniform Fourier sampling matrix, which performs Fourier transform on the non-integer grid points (NUFFT); *A* ^*h*^ stands for the conjugate transpose of *A*; *f* is the Fourier transform of acquired projections; $\left\| \cdot \right\|_{{L_{2} }}$ is an operator that calculates the Euclidean norm (*L*_2_-norm); *ɛ* is a control parameter that is determined empirically according to the noise level; $\left\| \cdot \right\|_{{L_{0} }}$ stands for the operator that calculates the number of the non-zero terms. $P$ is a diagonal sparse transformation matrix, whose diagonal element $\emptyset$ is defined as in Eq. 4.

4 $$\emptyset x = \emptyset \left( x \right)\mathop{=}\limits^{\text{def}} \left\{ {\begin{array}{*{20}c} {0,~(if \quad x < 0)} \\ {1, \left( {if \quad x \ge 0} \right)} \\ \end{array} } \right. .$$

The complete workflow of ICON can be divided into four steps (Deng* et al*. 2016):

Step 1: Pre-processing. Align tilt series and correct contrast transfer function (CTF).
Step 2: Gray value adjustment. Subtract the most frequently appeared pixel value in the micrographs, which is given from the embedding material (*e.g*., resin or vitrified ice).
Step 3: Reconstruction and pseudo-missing-validation. Reconstruct tilt series into a 3D volume with an iterative procedure of fidelity preservation and prior sparsity restriction, and evaluate the restored information with pseudo-missing-validation.
Step 4: Verification filtering. Exclude the incorrectly restored information.

A series of tests showed that Step 3 accounts for at least 95% of the execution time of ICON. Thus, the major task for accelerating ICON is parallelizing Step 3 effectively on GPU. Since the procedures of “reconstruction” and “pseudo-missing-validation” are similar, only the parallelization of “reconstruction” will be discussed in this paper.

The major steps of “reconstruction” can be briefly described as followed.

Step 3.1: Fidelity preservation step. In this step, steepest descent method (Goldstein 1965) is used to calculate the subject function of Eq. 3 as follows:

5 $$r = A^{h} WAx^{k} - A^{h} Wf,$$

6 $$\alpha = \frac{{r^{T} r}}{{r^{T} A^{h} WAr}},$$

7 $$y^{k + 1} = x^{k} - \alpha{r},$$

where *x* ^*k*^ is the 2D reconstructed slice of the *k*th iteration, *r* is the residual, *α* is the coefficient used to control the step of updating, *y* ^*k*+1^ is the intermediate updating result of the (*k* + 1)th iteration.

Step 3.2: Prior sparsity restriction step. The diagonal sparse transformation matrix *P* can be re-formulated as a “hard threshold”-like operation as in Eq. 8:

8 $$x^{k + 1} = H\left( {y^{k + 1} } \right) = \left\{ {\begin{array}{*{20}c} {0,} & {{\text{if}} \quad y^{k + 1} < 0} \\ {y^{k + 1} ,} & {{\text{if}} \quad y^{k + 1} \ge 0} \\ \end{array} } \right.,$$

where *y* ^*k*+1^ is the intermediate updating result of the (*k* + 1)th iteration. $H( \cdot )$ is a thresholding function, *x* ^*k*+1^ is the 2D reconstructed slice of the (*k* + 1)th iteration.

We classified the operations of these two steps into three types: (1) the summation of a matrix; (2) element-wise operations of matrices; (3) the NUFFT and the adjoint NUFFT. For a fast summation of matrix, we took advantage of the API function *cublasSasum* from the standard CUDA library cuBLAS (NVIDIA Corp, 2007). For type 1 and 2, parallel strategies are proposed in the following sections.

### Parallelizing element-wise operations of matrices

GPU is a massively multi-threaded data-parallel architecture, which contains hundreds of scalar processors (SPs) (Lindholm* et al*. 2008). NVIDIA provides the programming model on GPU called CUDA. The CUDA program running on GPU is called Kernel, which consists of thousands of threads. Thread is the basic running unit in CUDA programming model and it has a three-level hierarchy: grid, block, thread. Besides, CUDA devices use several memory spaces including global, shared, texture, and registers. Of these different memory spaces, global memory is the largest but slowest in data accessing. CUDA provides API function *cudaMemcpy* to transfer data between host memory and device memory; the time-consuming of such transfer sometimes is non-negligible especially for an iterative procedure like ICON reconstruction.

Since micrographs in ET are usually large (*e.g*., 2 k × 2 k or 4 k × 4 k in float or short format) and exceed the limitation of most types of CUDA device memory (*e.g*., 16 or 48 KB for shared memory), data in ICON-GPU are restored in global memory using float format. In order to cut down the time-consuming of memory transfer, we parallelized all operations of ICON on GPU even though some operations may have negligible speedups.

**Fig. 5:**
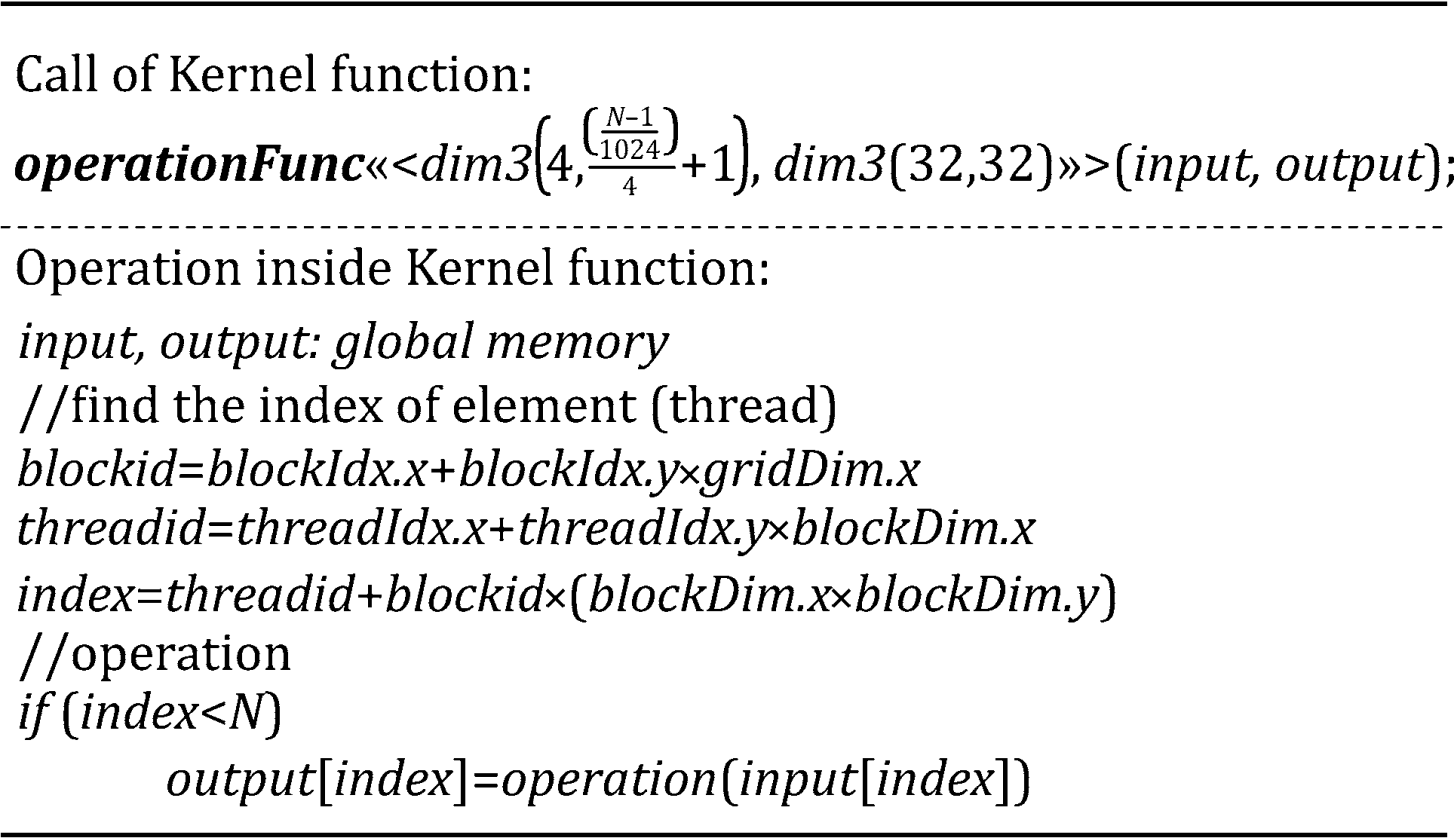
Pseudo codes for calling a kernel function and the operations inside a kernel function for element-wise operations

To deal with element-wise operation, ICON-GPU uses a 2D distribution of threads with a fixed block size of 32 × 32 and a fixed grid size of 4*β*, *β* is a parameter to be determined according to the matrix size *N*. ICON-GPU assigns the operation of one element to one thread according to the index of element. Pseudo codes for calling a kernel function and the operations inside a kernel function are shown in Fig. 5.

### Parallelizing NUFFT and adjoint NUFFT

First, we give a brief description of NUFFT. Given the Fourier coefficients $\hat{f}_{\varvec{k}} \in {\mathbb{C}},\varvec{k} \in I_{\varvec{N}}$ and $I_{\varvec{N}} = \left\{ {\varvec{k} = \left( {k_{t} } \right)_{t = 0, \ldots,d - 1} \in {\mathbb{Z}}^{d} : - \frac{{{\text{N}}_{t} }}{2} \le k_{t} < \frac{{{\text{N}}_{t} }}{2},\;t = 0, \ldots ,d - 1} \right\}$ as input, NUFFT tries to evaluate the following trigonometric polynomial efficiently at the reciprocal points $\varvec{x}_{j} \in \left[ { - \frac{1}{2},\frac{1}{2}} \right)^{d} ,\;\;\;j = 0, \ldots ,M - 1:$

9 $$f_{j} = f\left( {\varvec{x}_{j} } \right) = \mathop \sum \limits_{{\varvec{k} \in I_{\varvec{N}} }} \hat{f}_{\varvec{k}} e^{{ - 2\pi i\varvec{kx}_{j} }} , \;\;\;j = 0, \ldots ,M - 1.$$

Correspondingly, the adjoint NUFFT tries to evaluate Eq. 10 at the frequency *k*.

10 $$\hat{h}_{\varvec{k}} = \mathop \sum \limits_{j = 0}^{M - 1} f_{j} e^{{2\pi i\varvec{kx}_{j} }} .$$

NFFT3.0 (Keiner* et al*. 2010), a successful and widely used open source C library, is used in ICON-CPU for NUFFT and adjoint NUFFT. Yang* et al*. proposed a different theoretical derivation of NFFT and demonstrated the high efficiency of GPU acceleration of NFFT (Yang* et al*. 2015, 2016). To make ICON-GPU consistent with ICON-CPU, in this work, we parallelized the NUFFT and the adjoint NUFFT based on the algorithms described in NFFT3.0 and the algorithm of 2D NUFFT is displayed in Algorithm 1. *φ*(*x*) and $\hat{\varphi }\left( k \right)$ are the window functions. In this work, the (dilated) Gaussian window functions (Eqs. 11, 12) are used.

11 $$\varphi \left( x \right) = \left( {\pi b} \right)^{{ - \frac{1}{2}}} e^{{ - \frac{{\left( {nx} \right)^{2} }}{b}}} \;\;\;\left( {b = \frac{2\sigma }{2\sigma - 1}\frac{m}{\pi }} \right),$$

12 $$\hat{\varphi }\left( k \right) = \frac{1}{n}e^{{ - b\left( {\frac{\pi k}{n}} \right)^{2} }} ,$$

where *x* is a component of the reciprocal points $\varvec{x}$, *k* is a component of the frequencies $\varvec{k}$, *σ* is a component of the oversampling factors *σ* with *σ* > 1. In this work, *σ* = 2, *n* is one component of *n* = *σN*, $m \in {\mathbb{N}}$ and *m* ≪ *n*. In this work, *m* = 6.

The operations in 2D NUFFT and 2D adjoint NUFFT can be classified into three types: (1) element-wise operations of matrices; (2) 2D FFT; (3) calculation of window functions *φ*(*x*) and $\hat{\varphi }\left( k \right)$. The parallel strategy of type 1 is the same as the strategy described in Section “Parallelizing element-wise operations of matrices.” For type 2, to achieve a high performance FFT, we took advantage of the NVIDIA’s FFT library, CUFFT (NVIDIA Corp 2007). Since ICON is an iterative algorithm, 2D NUFFT and 2D adjoint NUFFT will be repeated many times. To cut down the time of calculation and memory transfer, we pre-computed the window functions for once and stored them in the device memory.

**Fig. 6:**
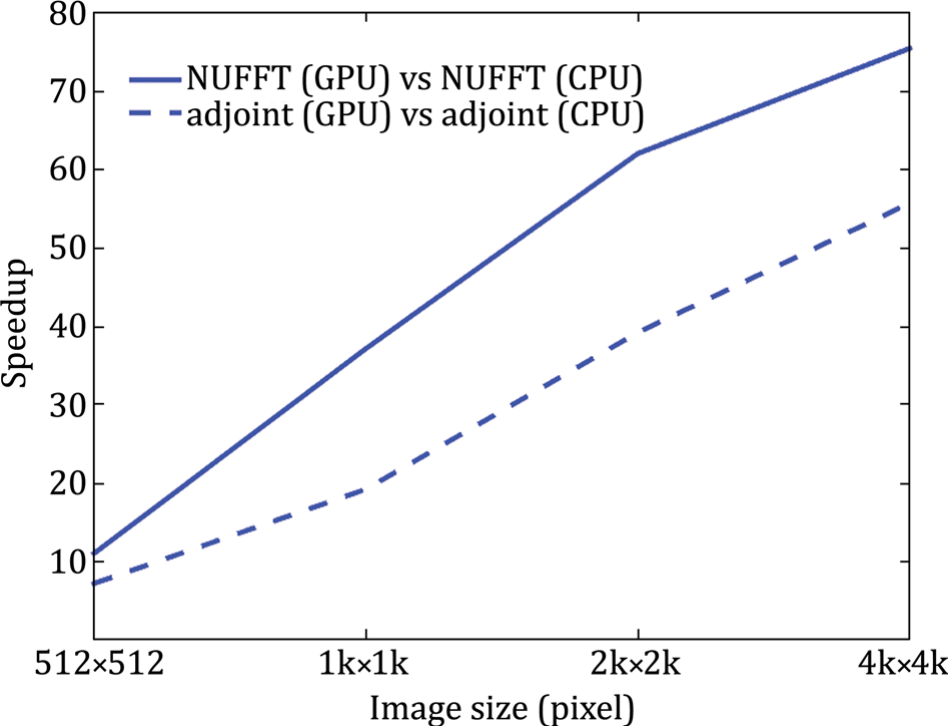
The speedups of parallel NUFFTs compared to NFFT3.0

Parallel NUFFTs were tested using a resin-embedded ET dataset (see “Resin embedded ET Dataset” for details). Here, all CPU programs ran on one core (thread) of an Intel^®^ Xeon™ CPU E5-2620 v2 @ 2.1 GHz (six cores per CPU) and all GPU programs ran on a NVIDIA Tesla K20c (2496 CUDA cores and 5 GB device memory). The accelerations of parallel NUFFTs improve when the image size increases and are up to 75.4x for NUFFT and 55.7x for adjoint NUFFT in the transform of one 4 k × 4 k image (Fig. 6).

### Resin-embedded ET dataset

We tested ICON-GPU using a resin-embedded ET dataset of MDCK cell section. The tilt angles of the dataset originally ranged from −68° to +68° with 1° increment. In order to verify ICON-GPU’s ability of restoring missing information, we extracted every other projection from the original dataset to generate a new tilt series with 2° increment for the following experiments. The tilt series were aligned using atom align (Han* et al*. 2014). The original image size is 4 k × 4 k with a pixel size of 0.72 nm. We also compressed the tilt series with factors of two, four, eight to generate datasets with smaller sizes of 2 k × 2 k, 1 k × 1 k, and 512 × 512, respectively.

## References

[CR1] Batenburg K, Sijbers J (2011). Dart: a practical reconstruction algorithm for discrete tomography. IEEE Trans Image Process.

[CR2] Carazo JM, Carrascosa JL (1987). Restoration of direct Fourier three-dimensional reconstructions of crystalline specimens by the method of convex projections. J Microsc.

[CR3] Castaño-Díez D, Kudryashev M, Arheit M, Stahlberg H (2012). Dynamo : a flexible, user-friendly development tool for subtomogram averaging of cryo-em data in high-performance computing environments. J Struct Biol.

[CR4] Chen Y, Förster F (2014). Iterative reconstruction of cryo-electron tomograms using nonuniform fast Fourier transforms. J Struct Biol.

[CR5] Chen Y, Zhang Y, Zhang K, Deng Y, Wang S, Zhang F, Sun F (2016). Firt: filtered iterative reconstruction technique with information restoration. J Struct Biol.

[CR6] Deng Y, Chen Y, Zhang Y, Wang S, Zhang F, Sun F (2016). Icon: 3d reconstruction with ‘missing-information’ restoration in biological electron tomography. J Struct Biol.

[CR7] Donoho DL (2006). Compressed sensing. IEEE Trans Inf Theory.

[CR8] Fernández JJ (2008). High performance computing in structural determination by electron cryomicroscopy. J Struct Biol.

[CR9] Fernández JJ, Carazo JM, García I (2004). Three-dimensional reconstruction of cellular structures by electron microscope tomography and parallel computing. J Parallel Distrib Comput.

[CR10] Fridman K, Mader A, Zwerger M, Elia N, Medalia O (2012). Advances in tomography: probing the molecular architecture of cells. Nat Rev Mol Cell Biol.

[CR11] Gilbert P (1972). Iterative methods for the three-dimensional reconstruction of an object from projections. J Theor Biol.

[CR12] Goldstein AA (1965). On steepest descent. J Soc Ind Appl Math.

[CR13] Goris B, Broek WVD, Batenburg KJ, Mezerji HH, Bals S (2012). Electron tomography based on a total variation minimization reconstruction technique. Ultramicroscopy.

[CR14] Han R, Zhang F, Wan X, Fernández JJ, Sun F, Liu Z (2014). A marker-free automatic alignment method based on scale-invariant features. J Struct Biol.

[CR15] Keiner J, Kunis S, Potts D (2010). Using NFFT 3—a software library for various nonequispaced fast Fourier transforms. ACM Trans Math Softw.

[CR16] Leary R, Saghi Z, Holland PAMDJ (2013). Compressed sensing electron tomography: theory and applications. Ultramicroscopy.

[CR17] Liao X, Xiao L, Yang C, Lu Y (2014). Milkyway-2 supercomputer: system and application. Front Comput Sci.

[CR18] Lindholm E, Nickolls J, Oberman S, Montrym J (2008). NVIDIA tesla: a unified graphics and computing architecture. IEEE Micro.

[CR19] Lučić V, Förster F, Baumeister W (2005). Structural studies by electron tomography: from cells to molecules. Annu Rev Biochem.

[CR20] Lučić V, Rigort A, Baumeister W (2013). Cryo-electron tomography: the challenge of doing structural biology *in situ*. J Cell Biol.

[CR21] Mersereau RM (1976). Direct Fourier transform techniques in 3-d image reconstruction. Comput Biol Med.

[CR22] NVIDIA Corp (2007) CUDA CUFFT Library

[CR23] Radermacher M, Frank J (1992). Weighted back-projection methods. Electron tomography.

[CR24] Rigort A, Villa E, Bäuerlein FJB, Engel BD, Plitzko JM (2012). Chapter 14—integrative approaches for cellular cryo-electron tomography: correlative imaging and focused ion beam micromachining. Methods Cell Biol.

[CR25] Saghi Z, Holland DJ, Leary R, Falqui A, Bertoni G, Sederman AJ, Gladden LF, Midgley PA (2011). Three-dimensional morphology of iron oxide nanoparticles with reactive concave surfaces. A compressed sensing-electron tomography (CS-ET) approach. Nano Lett.

[CR26] Saghi Z, Divitini G, Winter B, Leary R, Spiecker E, Ducati C, Midgley PA (2015). Compressed sensing electron tomography of needle-shaped biological specimens—potential for improved reconstruction fidelity with reduced dose. Ultramicroscopy.

[CR27] Sezan MI, Stark H (1983). Image restoration by convex projections in the presence of noise. Appl Opt.

[CR28] Yahav T, Maimon T, Grossman E, Dahan I, Medalia O (2011). Cryo-electron tomography: gaining insight into cellular processes by structural approaches. Curr Opin Struct Biol.

[CR29] Yang SC, Wang YL, Jiao GS, Qian HJ, Lu ZY (2015). Accelerating electrostatic interaction calculations with graphical processing units based on new developments of ewald method using non-uniform fast Fourier transform. J Comput Chem.

[CR30] Yang SC, Qian HJ, Lu ZY (2016) A new theoretical derivation of NFFT and its implementation on GPU. Appl Comput Harmon Anal. doi:10.1016/j.acha.2016.04.009

